# Genetic structure across urban and agricultural landscapes reveals evidence of resource specialization and philopatry in the Eastern carpenter bee, *Xylocopa virginica* L.

**DOI:** 10.1111/eva.13078

**Published:** 2020-08-28

**Authors:** Kimberly M. Ballare, Shalene Jha

**Affiliations:** ^1^ Department of Integrative Biology Biological Laboratories The University of Texas at Austin Austin TX USA; ^2^ Department of Ecology and Evolutionary Biology University of California Santa Cruz Santa Cruz CA USA

**Keywords:** human‐altered landscapes, human‐mediated dispersal, isolation by resistance, landscape genetics, nest‐site fidelity, pollinators

## Abstract

Human activity continues to impact global ecosystems, often by altering the habitat suitability, persistence, and movement of native species. It is thus critical to examine the population genetic structure of key ecosystemservice providers across human‐altered landscapes to provide insight into the forces that limit wildlife persistence and movement across multiple spatial scales. While some studies have documented declines of bee pollinators as a result of human‐mediated habitat alteration, others suggest that some bee species may benefit from altered land use due to increased food or nesting resource availability; however, detailed population and dispersal studies have been lacking. We investigated the population genetic structure of the Eastern carpenter bee, *Xylocopa virginica,* across 14 sites spanning more than 450 km, including dense urban areas and intensive agricultural habitat. *X. virginica* is a large bee which constructs nests in natural and human‐associated wooden substrates, and is hypothesized to disperse broadly across urbanizing areas. Using 10 microsatellite loci, we detected significant genetic isolation by geographic distance and significant isolation by land use, where urban and cultivated landscapes were most conducive to gene flow. This is one of the first population genetic analyses to provide evidence of enhanced insect dispersal in human‐altered areas as compared to semi‐natural landscapes. We found moderate levels of regional‐scale population structure across the study system (*G*ʹ_ST_ = 0.146) and substantial co‐ancestry between the sampling regions, where co‐ancestry patterns align with major human transportation corridors, suggesting that human‐mediated movement may be influencing regional dispersal processes. Additionally, we found a signature of strong site‐level philopatry where our analyses revealed significant levels of high genetic relatedness at very fine scales (<1 km), surprising given *X. virginica's* large body size. These results provide unique genetic evidence that insects can simultaneously exhibit substantial regional dispersal as well as high local nesting fidelity in landscapes dominated by human activity.

## 1 INTRODUCTION

Anthropogenic land use can dramatically alter ecological processes, often by changing habitat suitability and thus the persistence and movement of native species. One common result of intensive human land‐use practices, such as urbanization and cultivation, is the creation of homogenized altered habitats that may functionally isolate remnant habitat patches (Grimm et al., 2008; Saunders, Hobbs, & Margules, 1991). Such landscapes can lead to greater distances between suitable habitat patches which can then limit the dispersal of reproductive individuals (Athrey, Barr, Lance, & Leberg, 2012; Delaney, Riley, & Fisher, 2010; McRae, 2006; Templeton, Shaw, Routman, & Davis, 1990; Vandergast et al., 2009), creating isolated populations that may be vulnerable to deleterious effects of inbreeding and genetic drift (Bohonak, 1999; sensu Wright, 1948). Signatures of dispersal, inbreeding, and genetic drift may be detected in a population's genetic structure, which is defined by a species’ demographic history and the amount of gene flow between populations over many generations (Slatkin, 1985). Hence, measuring population genetic structure is often used to understand the rate of gene flow, providing proxy insight into a species’ colonization and dispersal patterns (Broquet & Petit, 2009) and potentially revealing the environmental factors that facilitate or impede movement across spatial scales.

While the overall number of studies investigating the impacts of landscape composition on species population genetics has increased in recent years (reviewed in Balkenhol, Cushman, & Storfer, 2015 and Storfer, Murphy, Spear, Holderegger, & Waits, 2010), most previous work has largely focused on vertebrates, primarily evaluating patterns at large spatial scales (>10 km). Very few studies have examined finer spatial patterns that could provide insight into the dispersal and nesting dynamics of smaller‐bodied but abundant and biodiverse taxa such as insects (Holyoak, Casagrandi, Nathan, Revilla, & Spiegel, 2008; Scheffers, Joppa, Pimm, & Laurance, 2012). This literature gap is especially important to consider given the establishment of international priorities to conserve insects that provide valuable ecosystem services such as pollination (IPBES, 2019), and the expansion of national efforts that target key insect pollinators, especially bees (Vilsack & McCarthy, 2015).

Recent studies have documented widespread declines of wild bee pollinators caused primarily by human‐mediated habitat alteration (Goulson, Nicholls, Botias, & Rotheray, 2015; Winfree, Bartomeus, & Cariveau, 2011). Bees may be uniquely vulnerable to landscape alteration because of potentially low underlying levels of genetic diversity due to their haplodiploid genetic systems (Zayed & Packer, 2005), and contemporary human‐altered land use has been shown to be a significant driver of genetic structure in several bee species (Davis, Murray, Fitzpatrick, Brown, & Paxton, 2010; Dreier et al., 2014; Jha, 2015; Suni & Brosi, 2011). However, past population genetic studies have primarily focused on bee taxa already believed to be sensitive to human‐mediated habitat change. In contrast, some bee species may exhibit equally high or higher abundance in anthropogenically disturbed landscapes assumedly because they are able to utilize resource patches within these altered habitats (Ballare, Neff, Ruppel, & Jha, 2019; Gardiner, Burkman, & Prajzner, 2013). For example, some large‐bodied bee species have been documented to heavily utilize human‐modified land use for foraging (e.g., mass flowering crops, Westphal, Steffan‐Dewenter, & Tscharntke, 2003; but see Larsen, Williams, & Kremen, 2005) and nesting sites (e.g., soil at field edges, Concepción, Díaz, & Baquero, 2007). However, it is not clear whether these habitats impede or facilitate dispersal and resulting gene flow, particularly for species with traits that typically promote movement, such as larger body size (Lopez‐Uribe, Jha, & Soro, 2019; Stow, Silberbauer, Beattie, & Briscoe, 2007).

Body size in particular is often hypothesized to correlate with low levels of genetic structure in many taxa including bees, because of the putative connection between organism size and maximum dispersal distances (Böhning‐Gaese, Caprano, van Ewijk, & Veith, 2006; Greenleaf, Williams, Winfree, & Kremen, 2007; Sekar, 2012; but see Castilla et al., 2017). Indeed, a recent meta‐analysis of 43 bee studies found that measures of population structure, including *F*_ST_ and *G*ʹ_ST_, significantly decreased with increasing bee body size (Lopez‐Uribe et al., 2019), with many larger species exhibiting little to no regional‐scale genetic structure (e.g., Lozier, Strange, Stewart, & Cameron, 2011) as compared to smaller species. Additionally, across body sizes, it is theoretically possible that the genetic effects of organism size and mobility can be compounded by fragmented landscapes that limit dispersal (Hanski, 2011), though this also depends on the resource utilization of the focal species.

Indeed, the nesting and foraging resources available to bees are highly dependent on human land management and can impact genetic structure at both the regional scale and finer spatial scales. Many bee species require distinct materials to construct their nests, including specific soil types, softwood, or pre‐existing cavities of the correct size (Michener, 2000). Because these resources may be patchily distributed across human‐altered landscapes (Cane, 2001), it is likely that altered habitat may be critical in mediating the dispersal and population persistence of nest‐specialized bee species. For example, past work examining the nest‐specialist species, *Colletes floralis*, found human‐modified land use to be more resistant to bee movement, likely due to increased coastal urbanization causing loss of specialist nesting habitat in sandy soil (Davis et al., 2010). Nest‐specialist species may also show strong philopatric tendencies, leading to population structure at small geographic scales, even if there are signals of substantial gene flow evident at larger scales (Franzén, Larsson, & Nilsson, 2007; Lopez‐Uribe, Morreale, Santiago, & Danforth, 2015; Schenau & Jha, 2016). It is difficult to directly measure these indices for highly mobile species, so indirect methods such as fine‐scale analyses of genetic spatial autocorrelation can be used to infer levels of nest‐site fidelity (e.g., Lopez‐Uribe et al., 2015; Stow et al., 2007).

In this study, we investigated both regional and fine‐scale population genetic structures in the Eastern carpenter bee *Xylocopa virginica* L. This species is a large carpenter bee native to southern and eastern North America, and is commonly found in both urban and rural areas across this range. Females exhibit solitary to semi‐social nesting behavior and construct their nests by excavating holes in sound wood (Michener, 2000), often in human‐associated materials including man‐made wooden structures, timber, and firewood (Balduf, 1962). Natural history accounts suggest that individual *X. virginica* may exhibit philopatry; they have been documented refurbishing nests of previous generations (Balduf, 1962; Chaves‐Alves et al., 2011) and may be “exceedingly slow at dispersing to new sites” (Balduf, 1962 p. 266), but these accounts have never been evaluated quantitatively or with genetic tools. In contrast, given *Xylocopa* species’ large body sizes (*X. virginica* intertegular distance (ITD, Cane, 1987) = 5.68 mm), large carpenter bees are believed to be long‐distance dispersers, especially in human‐altered landscapes, but this also has never been quantitatively evaluated (Cane, 2001). Urban and agricultural landscapes have been shown to be significant barriers of dispersal for other large‐bodied bees that nest underground (Davis et al., 2010; Jha & Kremen, 2013b), but because *X. virginica* nests above ground, often in man‐made structures, it is possible that human‐altered landscapes may actually facilitate their dispersal by increasing the number of potential nesting sites or by increasing human‐mediated dispersal through the movement of firewood or lumber.

In this study, we utilized highly polymorphic microsatellite loci in *X. virginica* to investigate population genetic structure across multiple scales covering more than 450 km of rapidly urbanizing and agriculturally intense habitat in the southern United States. Specifically, we investigated (a) levels of regional‐scale population structure, (b) signatures of regional genetic isolation by geographic distance (IBD) and isolation by resistance (IBR) across contemporary land use, and (c) levels of fine‐scale genetic structure. Specifically, we hypothesized that (a) *X. virginica* exhibits low levels of regional‐scale population genetic structure because of the species’ large body size and thus potential for long‐distance dispersal, (b) *X. virginica* populations experience greater genetic differentiation with increasing geographic distance but lower genetic differentiation across more urbanized and cultivated habitats, due to higher nesting availability in human‐altered landscapes, and (c) at local scales, *X. virginica* exhibits fine‐scale genetic structure indicative of high levels of nest‐site fidelity and philopatry.

## 2 MATERIALS AND METHODS

### 2.1 Specimen collection

Adult female *Xylocopa virginica* (*N* = 598) were collected from 33 sites across 14 regions that spanned a ~ 450 km corridor in central northern Texas between May and July in 2013 (Table S1). Regions were separated by at least 10 km, with the farthest sites in the study area separated by more than 450 km (mean = 210.27 km, *SD* = 145.25 km). Bees were collected within a 50 x 50 m plot established in an area with abundant floral resources (at a subset of sites described in Ballare, Neff, et al., 2019 and Ritchie, Ruppel, & Jha, 2016) Specimens were collected opportunistically in two ways: by hand netting for 1 hr within the plot and by passive trapping using unbaited blue vane traps suspended from a wooden stand located at the center of the plot (as per Stephen & Rao, 2007). Traps were empty of any preservative and left for 5 days at each site prior to collection. Empty blue vane traps have been shown to be preferable for field DNA preservation in dry weather conditions as compared to field preservation in propylene glycol (e.g., Ballare, Pope, et al., 2019). Netted specimens were stored frozen at −20°C, and trapped specimens were stored in 100% EtOH at 4°C prior to pinning and drying for specimen curation and species identification.

In order to test for fine‐scale population structure, five regions were selected for fine‐scale sampling. In these regions, four additional sites were sampled along a transect located at 300, 600, 900, and 1,200 m away from the original site; this established five evenly spaced study sites with similar land cover across each of the five points. Specimens were collected at each site after establishing a 50 × 50 m plot using the same sampling and preservation methods as described above.

### 2.2 DNA extraction, microsatellite amplification, and genotyping

Genomic DNA was extracted from a single hind leg of each dried specimen, using a modified DNAzol ® extraction protocol (Chomczynski, Mackey, Drews, & Wilfinger, 1997). We used the manufacturer's recommended protocol with volumes scaled to fit in a 96‐well plate format with a maximum well volume of 0.2 ml. Tissue was ground to a powder using a Mini‐BeadBeater‐96 (BioSpec) and 10 1.0 mm Zirconia/Silica beads per sample (BioSpec 11079110z) before proceeding with the remaining lysis and DNA extraction steps. DNA concentration was quantified using a NanoDrop 8,000 spectrophotometer and indicated no substantial difference between netted and trapped specimens.

Genomic DNA was amplified at 10 polymorphic microsatellite loci (Table S2) using the Qiagen Multiplex PCR Kit. We optimized 7 species‐specific markers described by Vickruck (2014), two markers developed for *X. frontinalis* (Augusto et al., 2012), and one marker developed for *X. grisescens* (Augusto et al., unpublished, GenBank Accession: KC168062). Markers were grouped into two multiplexes with each primer at 2 µM per mix, and forward primers contained a fluorescent tag (6‐FAM, VIC, NED, or PET) to detect individual markers during electrophoresis. Each multiplex was amplified in a 15 uL PCR using 7.5 µl of Qiagen 2x Multiplex PCR Master Mix, 1.5 µl primer mix, approximately 1–2 ng genomic DNA, and 3 µl of RNase‐free water. PCR conditions were the same for each multiplex: initial heat activation at 95°C for 15 min, then 30 cycles of 94°C for 30 s, 60.7°C for 90 s, and 72°C for 60 s, and a final extension step of 60°C for 30 min. Labeled PCR products were run on a 3,730 Sequencer (Applied Biosystems) at the Center for Biomedical Research Support DNA Sequencing Facility at the University of Texas at Austin. Alleles were called using GENEMARKER version 2.4.0 (SoftGenetics) and checked by eye. Thirty randomly chosen individuals (5%) were re‐genotyped to confirm accuracy of allele calls and to detect any genotype shifts or errors. Genotypes at all loci were found to be identical between runs.

### 2.3 Analyses

Although most carpenter bees are believed to be largely solitary species (Michener, 2000), this species has been documented to be facultatively social at the northern edge of its range (Richards & Course, 2015). Because the sociality of *X. virginica* is not known in Texas, we tested for the presence of full siblings in our dataset using COLONY v. 2.0.6.4 (Jones & Wang, 2010). Using the high precision method and assuming random mating, we detected the presence of 19 possible full siblings in the dataset and these were removed from further downstream analyses, making the full dataset a total of 579 nonsib individuals (Table S1 and Table S3).

### 2.4 Locus and population characteristics

We calculated basic allelic and population genetic statistics using the poppr package in R v 3.5.0 (Kamvar, Tab ima, & Grünwald, 2014). We performed tests for Hardy–Weinberg equilibrium (HWE) and linkage disequilibrium at each locus pair. We also calculated levels of observed and expected heterozygosity, as well as levels of inbreeding (*F*_IS_) within each population and locus. Effective population size (*N_e_*) of each site was estimated using COLONY 2.0.3.4 (Jones & Wang, 2010), using the full sib‐ship assignment method and assuming random mating. Transect locations were combined and analyzed as a single site for the COLONY analysis.

### 2.5 Regional‐scale population structure

We quantified levels of genetic structure using two different *F*‐statistics: *F*_ST_ (Nei, 1987) and the standardized index *G*ʹ_ST_ (Hedrick, 2005), which is more suitable for use with highly variable markers such as microsatellites (Meirmans & Hedrick, 2010). We calculated levels of global genetic structure in poppr (Kamvar et al., 2014) and calculated pairwise *F*_ST_ and *G*ʹ_ST_ between sites in GenAlEx v6.5 (Peakall & Smouse, 2006). We calculated these indices on a per region basis, where individuals collected at sites with multiple transect locations were analyzed together as a single site. Significance of these indices was evaluated by bootstrapping over loci for 9,999 permutations. As the pairwise *F*‐statistic is quite sensitive to missing data, it was necessary to remove individuals that had over 20% missing data for the pairwise analysis (*n* analyzed = 467; Table S3). The full dataset of 579 individuals was included in the global analysis.

To estimate the number of possible genetic groups (K) in the sampled populations, we used STRUCTURE v 2.3 (Pritchard, Stephens, & Donnelly, 2000). As in the *F*‐statistic calculations, we also analyzed individuals collected at regional transect locations together. However, as sampling was not even between sites and STRUCTURE can be biased by highly unequal population sizes (Puechmaille, 2016), we selected an equal number of random individuals from each transect location to have a maximum of 30 individuals per site (Table S3). Twenty runs of each K = 1 through K = 10 (200 total runs) were then performed with a burn‐in of 10,000 iterations followed by 1,000,000 Markov Chain Monte Carlo (MCMC) iterations using the default recommended model of correlated allele frequencies and admixture. We then used the output from these STRUCTURE runs to infer the true number of populations (K) using the Evanno method (Evanno, Regnaut, & Goudet, 2005) implemented in the program STRUCTURE Harvester (Earl & vonHoldt, 2011). This method infers the most likely K by calculating the rate of change in the log posterior probability across increasing K values. We additionally used the program CLUMPAK to visualize results (Kopelman, Mayzel, Jakobsson, Rosenberg, & Mayrose, 2015).

Because traditional Bayesian clustering programs can be prone to deducing artificial levels of population structure due to increasing geographic distance between collection points (Bradburd, Ralph, & Coop, 2013; e.g., Renner et al., 2016), we also inferred population clustering using the program GENELAND (Guillot, Mortier, & Estoup, 2005). GENELAND not only employs Bayesian clustering techniques in a similar way to STRUCTURE, but also incorporates the spatial coordinates of each site location in the model, returning the most probable number of genetic groups. We used the uncorrelated alleles model with the same range of possible populations as in the STRUCTURE analysis (K = 1 through 10). While the correlated allele model can sometimes detect more subtle population differentiation than other clustering algorithms, it is more sensitive to potential confounding effects of isolation by distance (Latch, Dharmarajan, Glaubitz, & Rhodes, 2006). We set the uncertainty of geographic coordinates to “0” and the null model to “true.” The final model was based on 1,000,000 MCMC iterations and a thinning value of 100. Five replicate runs were conducted to compare the similarity of results between models, as per Vickruck and Richards (2017). Finally, we further visualized genetic clustering by performing a principal component analysis (PCA) of individual genetic variation using the adegenet package (Jombart, 2008). Data were centered and scaled, with missing data set to mean (as per Malenfant, Davis, Cullingham, & Coltman, 2016). To visualize relatedness between sampling sites, we also generated a phylogenetic tree using the aboot function in the poppr package. We generated bootstrap support using 1,000 replicates using Nei's genetic distance between sites.

### 2.6 Regional‐scale patterns of genetic differentiation

To test the hypotheses of genetic structure based on geographic distance and land cover in the study region, we created a matrix of pairwise genetic distances for the full dataset of 579 individuals by calculating the Bruvo's genetic distance with the poppr package in R. Bruvo's distance (Bruvo, Michiels, D’souza, & Schulenburg, 2004) is a measure of genetic differentiation specific to microsatellite data that allows for missing data common in microsatellite datasets and takes into account the different numbers of repeat lengths and numbers of alleles per locus, improving resolution and accuracy compared with other genetic differentiation metrics such as Nei's distance (Grünwald, Everhart, Knaus, & Kamvar, 2017). To calculate the level of genetic differentiation explained by geographic distance alone (isolation by distance or IBD), we then performed multiple regression on distance matrices (MRDM) between the matrix of the mean pairwise Bruvo's distance per site and the matrix of pairwise geographic distances using the MRM function in the R package ecodist (Goslee & Urban, 2007) with 10,000 permutations. MRDMs are increasingly preferred over traditional Mantel tests due to lower occurrences of type I errors (Goulson et al., 2010; Jha & Kremen, 2013b). We conducted two IBD analyses (as in previous studies, Jha & Kremen, 2013b): one including all 34 collection points, with transect locations considered as separate sites, and one including 19 collection points, with only two sites per transect included (one site on each end of the 1.2 km transect), the latter specifically aimed to remove effects of natal philopatry. Geographic distances between each pair of sites were calculated using great‐circle distance which incorporates the curvature of the earth (van Etten, 2017).

In addition to testing for genetic IBD, we also tested for relationships between pairwise population genetic distance and contemporary land use, using circuit theory, or isolation by resistance (IBR, McRae, 2006). IBR evaluates environmental and/or geographic variation as predictors of genetic variation between populations, with the factors most limiting to gene flow considered “high resistance.” Although resistance can be calculated in a variety of ways, one of the fundamental approaches in landscape genetics is to develop resistance maps to test hypotheses based on landscape composition as well as predicted gene flow (Jaffé et al., 2015; Jha, 2015; Storfer et al., 2010). Therefore, we created multiple resistance surfaces based on results from past studies of other bee species and knowledge of *X. virginica* natural history. While there are no studies that investigate land‐use effects on gene flow in any wood‐nesting bee species, recent studies of bumblebees sampled across various land uses have shown that contemporary land use predicts gene flow significantly better than historic land use (Jha, 2015; Jha & Kremen, 2013a). Thus, we utilized the 2011 National Land Cover Database (NLCD) land‐use data (30‐m^2^ resolution, Homer, Fry, & Barnes, 2012) and created resistance maps to test groups of hypotheses (A, B, Set C) of effects of land use on *X. virginica* gene flow. First, we tested (A) the hypothesis that gene flow of *X. virginica* would be similarly limited by the same land uses that have been previously documented to limit gene flow in a number of bee species, specifically open water, impervious surfaces, and cropland (Goulson et al., 2010; Jha & Kremen, 2013b). As per Jha (2015), we first assigned open water, developed land, and cropland types a resistance value of 0.9, and grassland and forest types a resistance value of 0.1. However, as *X. virginica* is likely dependent on anthropogenic habitats in addition to forested habitats for nesting, we additionally tested another hypothesis (B) that urban, farmed, and forested habitats would have lower resistance to gene flow than open grassland habitats which contain little woody substrate. In this case, we assigned developed land, cultivated land, and forest habitats a resistance value of 0.1 and all other land cover types within the study area a value of 0.9. Finally, we tested a set of hypotheses (Set C) that single land uses are independently driving gene flow, with each of the land‐use types in Hypothesis B as single terms, with each assigned a resistance value of 0.1, and all other land‐use types assigned a resistance value of 0.9 (Jaffé et al., 2019; as per Jha, 2015). To test for any nonlinear relationships between genetic distance and land use, we also created three additional resistance maps per hypothesis, varying the low and high resistance values between 0.1–0.3 and 0.5–0.9, respectively (sensu Jha & Kremen, 2013b; Ortego, Aguirre, Noguerales, & Cordero, 2015; Zellmer & Knowles, 2009).

We calculated pairwise land‐use resistance distances (McRae, 2006) between all 34 sampling locations for all of our hypothesis‐driven maps (A, B, Set C) with the R package gdistance (van Etten, 2017) and used the raster package (Hijmans et al., 2015) to aggregate pixels to the mean value within 210 m^2^ (as per Jaffé et al., 2016, 2019) to account for the limited size of the study region, the short distances between transect sites (300 m), and the high levels of nest‐site fidelity presumed for this species. MRDMs were used to test for the relationship between calculated resistance distance matrices and mean Bruvo's genetic distances per site. We constructed models including geographic distance and land‐use resistance as independent variables (22 total models).

### 2.7 Fine‐scale spatial population structure

To assess any genetic signatures of site‐level philopatry, fine‐scale spatial population genetic structure was examined using the program SPAGeDi 1.5a (Hardy & Vekemans, 2002). All individuals in the main dataset (*n* = 579) were included in this analysis. Pairwise geographic distances between individuals were calculated using Loiselle's F_ij_ (Loiselle, Sork, Nason, & Graham, 1995) and binned in intervals that maximized evenness of pairs between the subregion (transect) sites. The binning intervals were as follows: 0 to 250 m, 250 to 500 m, 500 to 750 m, and 750 m to 1 km. The remaining individuals that were located at a greater distance than 1 km were combined in a single bin at a distance of 25 km. The number of pairs in each interval was > 2,500. Standard errors were computed by jackknifing over loci, and 95% confidence intervals were computed by permuting over genotypes and location with 10,000 permutations.

## 3 RESULTS

### 3.1 Locus and population characteristics

We scored 148 total alleles at 10 loci (mean 14.8 ± 2.93; Table S2). Using the genotype_curve function in poppr, we found 10 loci more than sufficient to distinguish individuals, with the accumulation curve saturating at 5–6 loci. After testing for departure from Hardy–Weinberg equilibrium (HWE) at each locus and site pair (330 total tests), we found six significant results after Bonferroni's correction, but no locus was consistently out of HWE across all sites. There were no null alleles found nor any linkage disequilibrium detected between any pair of loci, and thus, all loci were retained for further analyses.

We detected private alleles at nine of the sampled sites, with the WQ site containing the highest number of total private alleles (11, Table S1). Observed heterozygosity ranged from 0.449 to 0.686, and expected heterozygosity ranged from 0.300 to 0.715. Mean levels of inbreeding calculated by *F*_IS_ were relatively low (0.097 ± 0.010), but similar to *F*_IS_ levels observed in another recent study of *X. virginica* (Vickruck & Richards, 2017). One site at the southern edge of the study area (BC) showed somewhat higher levels of *F*_IS_ (0.210, Table S1). Estimated effective population sizes (*N_e_*) were also low, and ranged from 41 at site RRL and 144 at both HRM and RCR, although some sites had an estimated N_e_ of infinity, likely due to low sample sizes at those sites (as described in Jones & Wang, 2010).

### 3.2 Regional‐scale population structure

Global *F*_ST_ and *G*ʹ_ST_ values indicated moderate levels of regional‐scale population structure (*F*_ST_ = 0.042, *G*ʹ_ST_ = 0.146). Examination of pairwise *F*_ST_ and *G*ʹ_ST_ across sites indicated significant differentiation at all *G*ʹ_ST_ comparisons (Table S4), although several values were negative, indicating that the true *G*ʹ_ST_ value is 0 in these cases. Several *F*_ST_ comparisons were not significant, most often at sites with low sample sizes (Table S4).

**Figure 1:**
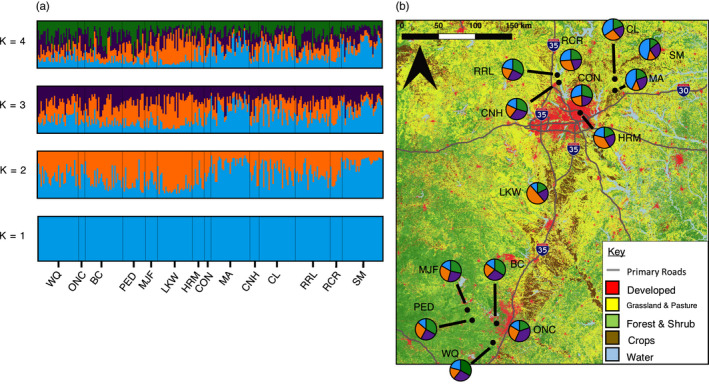
STRUCTURE results for *Xylocopa virginica* at all 14 collection sites. A subsample of random individuals were selected from transect locations and analyzed together as a single site for sites BC, CL, MA, SM, and WQ, each having a maximum of *n* = 30 individuals included in the analysis. (a) Population membership as computed by STRUCTURE for K = 1, K = 2, K = 3, and K = 4 from 10 microsatellite loci and visualized using CLUMPAK. Sites are arranged left to right by increasing latitude. Each vertical bar represents the probability that an individual genotype can be assigned to a particular population, indicated by different colors. Blue represents membership to genetic population 1, orange to genetic population 2, purple to genetic population 3, and green to genetic population 4. K = 4 was the most likely number of genetic populations (Evanno et al., 2005). (b) Map of collection sites displaying pie charts of mean population membership for K = 4 genetic populations. Segments are color‐coded by population membership as in the STRUCTURE plots (a)

We found that the likely number of K genetic populations was not completely consistent between GENELAND and STRUCTURE analyses. STRUCTURE analyses revealed that K = 4 was the most likely number of genetic populations by using the Evanno method to determine K (Evanno et al., 2005). However, after running GENELAND, results indicated that K = 1 was the most likely number of genetic populations in our study (Figure S1), suggesting that little distinct genetic clustering could be seen across our study region. Additionally, the PCA method could not distinguish clusters of distinct genetic groups and bootstrap support for distinct genetic groups in the phylogenetic tree tended to be low (~55% or less). Indeed, visualization of the STRUCTURE results indicates that individuals are highly admixed among genetic populations (Figure 1); this combined with the GENELAND and PCA results further suggests that highly distinct genetic clusters could not be detected within the sampling region (Figures S1 and S2a). Sites that had the lowest amounts of admixture in the STRUCTURE analysis had the highest probability of assignment in either genetic population 1 or population 2 in all Ks tested. These were located in the central (LKW) or northwestern (MA, SM) regions of the sampling area (Figure 1). This moderate distinction was also reflected in the phylogenetic tree, with SM and MA clustering together in a distinct clade from the other sites in 52% of trees (Figure S2b).

### 3.3 Regional‐scale patterns of population genetic differentiation

**Figure 2:**
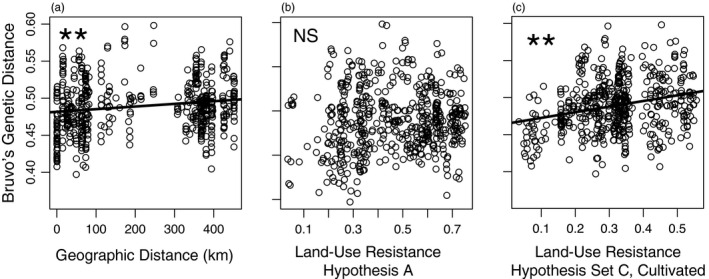
(a) Isolation by distance (IBD), and (b, c) isolation by resistance (IBR) for two resistance maps (Hypothesis A and Hypothesis C, cultivated; Table 1) among 34 sampling locations. Each point represents the average pairwise genetic distance (Bruvo et al., 2004) between sample sites. ** indicates significance at the α < 0.01 level for the MRDM models

When all 34 sites were included, MRDM models showed significant support for IBD (*p* = .009). When only 19 sites were included in the IBD analysis, we also found significant IBD, but the F and p values were much lower than for the model with all sites included (Table 1; Table S5). MRDM models showed the strongest support for IBR when developed land and cropland had lower resistance values and semi‐natural land had higher resistance values (Hypothesis B, Set C (developed and cultivated only)), although R^2^ values were relatively small across all models (Table 1; Figure 2; Table S5). There was not significant support for the model used in the past for bumblebees (Jha, 2015) (grassland and forest resistance = 0.1, all other classes = 0.9; *p* = .256). Models including forest cover at resistance value = 0.3 (but not 0.1) were also significant (Table S5). The best overall models were isolation by resistance with cultivated land as a single variable set to a lower resistance value (Set C, cultivated only, Table 1; Table S5).

**Table 1:** MRDM results investigating geographic distance, and resistance distances for five land‐use resistance surfaces (Hypotheses A, B, and Set C) and their relationship to average Bruvo's genetic distance per site (*N* = 528 pairs). Significant models are highlighted in bold. For results of all 22 models tested, see Table S5

Hypothesis	Land cover classes set to lower resistance (0.1–0.3)	Resistance value per land cover type					MRDM results		
		Developed	Cultivated	Forest and shrub	Grassland	Other classes^a^	*F*	*R* ^2^	*p*‐value
Geographic distance (all sites included)	NA	NA	NA	NA	NA	NA	**15.561**	**0.028**	**.009**
Geographic distance (sites > 1 km apart)	NA	NA	NA	NA	NA	NA	**4.011**	**0.023**	**.042**
A	Grassland and forest	0.9	0.9	0.1	0.1	0.9	4.265	0.008	.256
B	Developed, cultivated, and forest	0.1	0.1	0.1	0.9	0.9	**19.452**	**0.035**	**.008**
Set C	Developed only	0.1	0.9	0.9	0.9	0.9	**18.114**	**0.033**	**.017**
	Cultivated only	0.9	0.1	0.9	0.9	0.9	**38.810**	**0.068**	**.001**
	Forest only	0.9	0.9	0.1	0.9	0.9	8.111	0.015	.109

^a^ Other classes made up less than 5% of total land cover within the study region and included open water, barren, and wetlands, presumed noncritical bee habitat.

### 3.4 Fine‐scale spatial population structure

**Figure 3:**
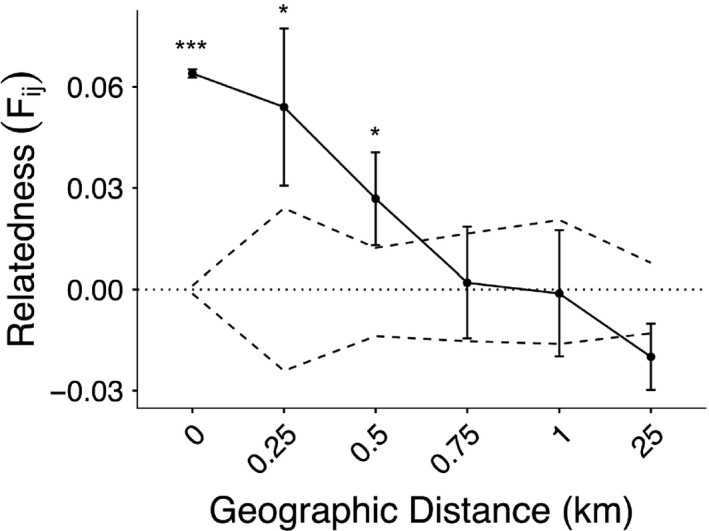
Spatial autocorrelation of genetic relatedness (Loiselle's *F_ij_*) versus geographic distance. The solid black line represents the mean of all pairwise relatedness coefficients between individuals at different distance intervals. Dashed lines represent permuted 95% confidence intervals (CI) for the null hypothesis that there was no correlation between relatedness and distance (*F_ij_* = 0). Upper and lower CI values at distance 0 are small but do not equal zero. Individuals at distances lower than 0.75 show significantly higher genetic relatedness than expected by random mating. * indicates significance at α < 0.05, and *** indicates significance at α < 0.001

We found evidence for significant fine‐scale spatial autocorrelation in genetic relatedness in *X. virginica* at the within‐site scale (*F_ij_* = 0.064, *p* < .001), the 0‐ to 0.25‐km scale (*F_ij_* = 0.054, *p* = .013), and the 0.25‐ to 0.5‐km scale (*F_ij_* = 0.026, *p* = .021), where individuals sampled within these distances were significantly more related to each other than expected at random. Average genetic relatedness declined as distance increased (Figure 3).

## 4 DISCUSSION

In this study, we utilized population genetic structure as an indirect but powerful measure of gene flow to provide evidence of both high regional dispersal and high natal site fidelity for a nest‐specialist pollinator inhabiting human‐dominated landscapes. We also found evidence for significant genetic isolation by distance (IBD), and even stronger support for significant genetic isolation by land‐use resistance (IBR), where our results indicated that human‐altered habitats are likely the most conducive habitats for *X. virginica* gene flow. Although past studies have indicated that some bee species exhibit high levels of dispersal across fragmented agricultural landscapes, similar to dispersal rates across semi‐natural landscapes (Herrmann, Westphal, Moritz, & Steffan‐Dewenter, 2007; Jaffé et al., 2015; Suni & Brosi, 2011), to our knowledge, this study represents the first line of evidence that urban and cultivated habitat can mediate enhanced dispersal for any native pollinator species.

### 4.1 Land‐use analyses suggest high IBR in comparison with IBD

We found significant IBR across the study area, with our best models predicting lower population structuring across urban and cultivated habitats. While we acknowledge that population structure describes both demographic history and microevolutionary processes (Gronau, Hubisz, Gulko, Danko, & Siepel, 2011), it can also provide unique insight into critical dispersal and colonization processes (Bohonak, 1999; Broquet & Petit, 2009). Specifically, our results provide evidence that urban and cultivated areas are mediating elevated levels of gene flow for *X. virginica*, either through human‐facilitated or natural dispersal. These results contrast previous pollinator studies investigating landscape drivers of genetic structure which have suggested that urban and cropland use can be highly restrictive to gene flow (Davis et al., 2010; Jha, 2015). However, these past studies have primarily investigated bee species that nest underground, which are therefore expected to be sensitive to the increases in impervious cover, soil impactions, or tilling associated with human‐altered landscapes.

Across taxa, there are very few studies which directly investigate genetic structuring in species predicted to exhibit greater dispersal in human‐altered landscapes (reviewed in LaPoint, Balkenhol, Hale, Sadler, & van der Ree, 2015; Storfer et al., 2010), and lower genetic structuring in urban and cultivated landscapes has never been explicitly documented for a native animal pollinator. However, this observed pattern was largely in line with our expectations, given that *X. virginica* is a frequent nester in wooden built structures in both urban and agricultural landscapes (e.g., houses, barns, fence posts, Gerling & Hermann, 1978). Cultivated areas may be especially predictive of lower genetic structure for this species, due to abundant nesting resources within wooden substrates in pastures and along field margins (Steffan‐Dewenter & Leschke, 2003), and ease of foraging in pasture and croplands (Holzschuh, Steffan‐Dewenter, Kleijn, & Tscharntke, 2007; Westphal et al., 2003), which may also facilitate dispersal. Indeed, some studies of bees in cultivated landscapes have found evidence that agricultural land facilitates gene flow to the same degree as natural landscapes (e.g., Jaffé et al., 2015, 2016), and a study of several related Brazilian *Xylocopa* species showed yearly increasing population densities within floral‐rich cultivated areas that had higher levels of nesting sites (Yamamoto, Junqueira, Barbosa, Augusto, & Oliveira, 2014). In our study, we found that natural forested landscapes were also significant drivers of *X. virginica* gene flow, but to a lesser extent than either urban or cultivated landscapes. This pattern has rarely been seen in insect studies, but one study of an endangered damselfly (*Coenagrion mercuriale*) suggested that open agricultural landscapes enhanced gene flow more than presumed preferred stream habitats, where natural forested habitats acted as barriers (Keller, van Strien, & Holderegger, 2012). In community‐level bee studies including *X. virginica*, nesting densities may increase in human‐altered areas with closer proximity to wooded landscapes, also highlighting the importance of conserving natural areas for pollinator habitat (Antonini, Martins, Aguiar, & Loyola, 2012; Ballare, Neff, et al., 2019; Greenleaf & Kremen, 2006). Overall, our study adds to a growing body of literature indicating that human‐altered landscapes, including both urban and cultivated habitats, may facilitate rather than hinder gene flow of many species.

Although weaker than the IBR results, we also documented significant genetic isolation by geographic distance (IBD) across the entire study area. Geographic IBD results from genetic drift differentiating allele frequencies at a faster rate than dispersal and gene flow can normalize them (Rousset, 1997); IBD has been documented for taxa across a wide range of body sizes and life‐history strategies (Meirmans, 2012). In past reviews, IBD has been shown to be more pronounced in species that have low levels of dispersal (Aguillon et al., 2017). Although some level of IBD is often found in population genetic studies (Bradburd et al., 2013; Storfer et al., 2010), it was surprising to find significant IBD in our study, especially within a large‐bodied insect. Significant IBD patterns previously observed in this and other bee species were presumed to be due to limited dispersal related to local resource requirements (Jaffé et al., 2016; Vickruck & Richards, 2017). Interestingly, the IBD signature found in our study was much weaker when sites within 1 km were excluded, indicating that while landscape‐scale IBD is present in *X. virginica*, much of the pattern is driven by high genetic relatedness at very fine scales.

### 4.2 Fine‐scale population structure as evidence of high philopatry

We document fine‐scale spatial genetic structure at least 10 times greater than values measured in previous studies of other bee species. Specifically, while we expected to see moderate levels of fine‐scale genetic structure, the magnitude of our observed relatedness values at local sites (*F_ij_* ≅ 0.03–0.06) and at spatial scales as small as 0.25 km was astonishingly high compared with similar studies in bees. For example, Lopez‐Uribe et al. (2015) also found significant spatial autocorrelation of the common plasterer bee *Colletes inaequalis* at similarly small spatial scales (<0.5 km), but the genetic relatedness for *C. inaequalis* was an order of magnitude lower than found within our study (*C. inaequalis* F_ij_ = 0.004). Studies of other bee species have found similar relatedness levels to our results (F_ij_ = 0.05–0.06), but at much larger spatial scales (5–50 km, Jaffé et al., 2016; Schenau & Jha, 2016). While it is possible that this high relatedness is simply due to dispersal limitation, given the observed long‐distance foraging activities of this and related species, often beyond 5km (Pasquet et al., 2008), our observed signatures of fine‐scale spatial genetic structure suggest the existence of philopatry related to the distinctive nesting specialization of *X. virginica*. Past studies have shown that pollinators which are foraging resource specialists show higher levels of population genetic structure than generalist foragers (Dellicour, Michez, Rasplus, & Mardulyn, 2015; Dellicour et al., 2017; Zayed et al., 2005; but see Lopez‐Uribe et al., 2019). While *X. virginica* is a forage generalist, our results are consistent with the hypothesis that nesting specialists may show similar spatial genetic structure patterns to specialist foragers due to high levels of natal site fidelity.

### 4.3 Evidence for moderate regional‐level population structure, but little genetic clustering

In our study, levels of global *G*ʹ_ST_ and *F*_ST_ were much higher than what we originally expected given *X. virginica's* large body size, especially since large‐bodied bees tend to have lower levels of population structure as compared to smaller‐bodied bees (as reviewed in Lopez‐Uribe et al., 2019). This general pattern is assumed to be a result of the longer‐distance flying and foraging abilities of large‐bodied bees (Greenleaf et al., 2007), which could correlate with greater dispersal abilities (Sekar, 2012; Stevens et al., 2014). For example, population structure for a number of bumblebee species with similar or smaller body sizes than *X. virginica* indicates much lower genetic structure at similar geographic scales to our study (e.g., Charman, Sears, Green, & Bourke, 2010; Lozier & Cameron, 2009). Instead, we document differentiation levels for *X. virginica* that are more similar to smaller‐bodied specialist bee species, such as the solitary foraging specialists *Andrena fuscipes* (ITD = 2.76, *G*ʹ_ST_ = 0.159, Exeler, Kratochwil, & Hochkirch, 2010) and *Euglossa championi* (ITD = 3.20, *G*ʹ_ST_ = 0.150, Suni, Bronstein, & Brosi, 2014). Our results add to the increasing body of evidence that large body size does not necessarily translate to low genetic structure for bees (Jaffé et al., 2016; Knight et al., 2005), as has been documented across other animal taxa. For example, ecological traits and habitat availability are often better predictors of genetic structure than body size in other highly mobile organisms such as butterflies (Stevens et al., 2013), fish (Carlsson, Olsen, Nilsson, Overli, & Stabell, 1999; Dalongeville, Andrello, Mouillot, Albouy, & Manel, 2016) and birds (Eo, Doyle, & DeWoody, 2010). In addition, although we did not sample males for this study, it is possible that *X. virginica* also exhibits sex‐biased genetic dispersal patterns. Observational and mark–recapture studies of *X. virginica* and related *Xylocopa* species suggest that both males and females remain close to natal sites during the reproductive season and over winter (Barthell, Reidenbaugh, & Griffith, 2006; Peso & Richards, 2011), but future genetic analyses of males would be useful in determining whether males are contributing more to longer‐distance dispersal than females.

Our Bayesian clustering analyses revealed weak but intriguing genetic clustering patterns within the sampling area, with the most likely K between K = 1 and K = 4 depending on the analysis program used. Most notably, the most distinct populations were located in the center (site LKW) and northeastern edge (sites MA and SM) of the study region in very rural areas. The remaining sites are more closely clustered around the urban areas of Austin and Dallas, and had higher levels of admixture, indicating that there is more genetic mixing between these sites despite being located further apart. This might be explained partially by the fact that most of the sites in the study are located within 70 km or less to the same major highway, the Interstate 35 corridor, one of the largest freight flows in the United States (U.S. Department of Transportation, 2020).

In contrast, MA and SM are more closely located to the less‐traveled Interstate 30 (see Figure 1); this suggests that the other more admixed sites might be a result of activity on Interstate 35 such as human‐mediated transport of wooden materials. While it is unclear whether the mechanism behind the patterns observed in our study system is due to human‐mediated or natural dispersal, we posit that it is likely a combination of both, given past documentation of unintentional bee movement (e.g., Gibbs & Sheffield, 2009; Portman, Burrows, Griswold, Arduser, & Cariveau, 2019) and frequent natural colonization of human‐associated structures by carpenter bees (Chaves‐Alves et al., 2011). The role of roadways in mediating genetic clustering has been documented in many taxa, although the majority of studies are focused on roads as barriers to gene flow (Balkenhol & Waits, 2009; Holderegger & Di Giulio, 2010). However, roads have been shown to increase abundance and genetic connectivity of many invasive and pest species (Handley et al., 2011; Meunier & Lavoie, 2012; Miles, Johnson, Dyer, & Verrelli, 2018; Pauchard & Alaback, 2004; Tang, Low, Lim, Gwee, & Rheindt, 2018), including through unintentional transport in wooden materials (Kerdelhué, Boivin, & Burban, 2014). The idea that road networks may act as “hubs” of gene flow for certain species is beginning to be investigated (Miles, Dyer, & Verrelli, 2018); however, the extent to which human‐altered environments can serve as facilitators of gene flow for nonpest and native species is still poorly understood.

### 4.4 Conservation implications

There have been many recent reports of wild bee declines across the globe, most often linked to human‐induced habitat change (Cameron et al., 2011; Goulson et al., 2015). In order to develop effective conservation strategies for bees and their associated pollination services, genetic tools are critical for understanding the demographic history and potential drivers of genetic structure (reviewed in López‐Uribe, Soro, & Jha, 2017). In this study, we show that the Eastern carpenter bee *Xylocopa virginica* displays both regional and localized levels of population genetic structure related to their nesting resource specialization and exceptionally high levels of natal site fidelity. Although this fine‐scale genetic relatedness and significant IBD indicate somewhat limited dispersal in this species, isolation‐by‐land‐use analyses reveal that urban, agricultural, and woody landscapes may facilitate *X. virginica* gene flow across the study region. Overall, these results suggest that there may be positive implications for the conservation of pollination services both locally and globally, as the nearly 400 *Xylocopa* species are important pollinators for both crops and native plants throughout the world (Chaves‐Alves et al., 2011; Junqueira, Hogendoorn, & Augusto, 2012; Sampson, Danka, & Stringer, 2004). As human alteration of natural landscapes continues, *X. virginica* and other wood‐nesting pollinators will likely benefit, reinforcing wild pollination services in both urban and agricultural areas.

## 5 Data Archiving Statement

Individual microsatellite genotypes, individual sample IDs, and DNA extraction plate information are deposited as a.csv file on DRYAD, https://doi.org/10.7291/D1Z39R

## Supporting information

Supplementary Material
