# Supplementary material for: Genetic structure across urban and agricultural landscapes reveals evidence of resource specialization and philopatry in the Eastern carpenter bee, Xylocopa virginica L
Source: Evol Appl. 2020 Aug 28;14(1):136–49. doi: 10.1111/eva.13078 (PMC7819568; doi:10.1111/eva.13078)
Supplement: Supplementary file 1 — Supplementary Material [file EVA-14-136-s001.pdf]

Supporting Information for "Genetic structure across urban and agricultural landscapes reveals evidence of resource specialization and philopatry in the Eastern carpenter bee, *Xylocopa virginica* L."

Kimberly M. Ballare[1,2] and Shalene Jha[1]

[1] Department of Integrative Biology, The University of Texas at Austin, 205 W 24th Street, 401 Biological Laboratories, Austin, TX 78712, USA

[2] Present Address: Department of Ecology and Evolutionary Biology, University of California Santa Cruz, Santa Cruz, CA 95060, USA

Table S1. Collection locations, coordinates, and genetic characteristics for 579 female *Xylocopa virginica* genotyped at 10 microsatellite loci. Some populations' N_e was calculated as infinite, likely due to small samples sizes in those populations (Jones & Wang 2010).

Site Name	Transect Number	Region (Nearest City)	Site Code	Latitude	Longitude	N	N_p	H_o	H_e	F_{IS}	N_e
Barton Creek Habitat Preserve	1	Austin	BC1	-97.915	30.306	18	0	0.555	0.632	0.123	-
Barton Creek Habitat Preserve	2	Austin	BC2	-97.916	30.303	10	0	0.449	0.600	0.253	-
Barton Creek Habitat Preserve	3	Austin	BC3	-97.912	30.300	0	-	-	-	-	-
Barton Creek Habitat Preserve	4	Austin	BC4	-97.910	30.298	13	0	0.516	0.622	0.171	-
Barton Creek Habitat Preserve	5	Austin	BC5	-97.909	30.296	11	0	0.478	0.631	0.243	-
Barton Creek Habitat Preserve	All	Austin	BC	-	-	52	0	0.509	0.645	0.211	61
Clymer Meadow Preserve	1	Dallas	CL1	-96.242	33.302	1	0	0.600	0.300	NA	-
Clymer Meadow Preserve	2	Dallas	CL2	-96.243	33.305	16	1	0.628	0.669	0.061	-
Clymer Meadow Preserve	3	Dallas	CL3	-96.243	33.308	5	0	0.502	0.565	0.112	-
Clymer Meadow Preserve	4	Dallas	CL4	-96.243	33.311	7	0	0.586	0.598	0.020	-
Clymer Meadow Preserve	5	Dallas	CL5	-96.243	33.314	8	0	0.553	0.655	0.157	-
Clymer Meadow Preserve	All	Dallas	CL	-	-	37	0	0.587	0.691	0.151	44
Clear Creek Natural Heritage	NA	Dallas	CNH	-97.065	33.261	7	1	0.614	0.651	0.056	∞
Connemara Prairie	NA	Dallas	CON	-96.703	33.083	5	0	0.572	0.550	-0.040	∞
Harry S Moss Park	NA	Dallas	HRM	-96.748	32.893	9	1	0.594	0.642	0.075	144
Lake Whitney State Park	NA	Waco	LKW	-97.355	31.925	26	2	0.589	0.676	0.130	44
Mathew's Prairie	1	Dallas	MA1	-96.237	33.172	6	0	0.592	0.653	0.093	-
Mathew's Prairie	2	Dallas	MA2	-96.238	33.170	10	0	0.620	0.622	0.003	-
Mathew's Prairie	3	Dallas	MA3	-96.239	33.168	36	1	0.588	0.639	0.080	-
Mathew's Prairie	4	Dallas	MA4	-96.241	33.167	26	2	0.623	0.653	0.046	-
Mathew's Prairie	5	Dallas	MA5	-96.242	33.165	22	1	0.606	0.698	0.132	-
Mathew's Prairie	All	Dallas	MA	-	-	100	4	0.604	0.687	0.121	92
M & J Farms	NA	Austin	MJF	-98.331	30.457	9	0	0.651	0.684	0.048	90
Onion Creek Wildlife Sanctuary	NA	Austin	ONC	-97.614	30.198	5	0	0.640	0.674	0.050	∞
Pedernales Falls State Park	NA	Austin	PED	-98.259	30.332	17	1	0.518	0.642	0.193	78
R & C Dairy	NA	Dallas	RCR	-96.910	33.531	9	0	0.629	0.696	0.097	144
Ray Roberts Lake State Park	NA	Dallas	RRL	-97.091	33.352	26	1	0.580	0.662	0.123	41
Smiley Woodfen Prairie	1	Dallas	SM1	-95.698	33.646	19	0	0.684	0.715	0.044	-
Smiley Woodfen Prairie	2	Dallas	SM2	-95.698	33.648	29	0	0.638	0.677	0.058	-
Smiley Woodfen Prairie	3	Dallas	SM3	-95.698	33.652	26	0	0.568	0.627	0.093	-
Smiley Woodfen Prairie	4	Dallas	SM4	-95.698	33.654	40	5	0.612	0.653	0.063	-
Smiley Woodfen Prairie	5	Dallas	SM5	-95.698	33.657	13	1	0.626	0.655	0.044	-
Smiley Woodfen Prairie	All	Dallas	SM	-	-	127	6	0.609	0.701	0.137	108
Water Quality Protection Lands	1	Austin	WQ1	-97.957	30.066	29	0	0.616	0.650	0.052	-
Water Quality Protection Lands	2	Austin	WQ2	-97.957	30.065	25	1	0.627	0.708	0.114	-
Water Quality Protection Lands	3	Austin	WQ3	-97.957	30.063	35	3	0.676	0.693	0.024	-
Water Quality Protection Lands	4	Austin	WQ4	-97.957	30.060	32	5	0.600	0.692	0.133	-
Water Quality Protection Lands	5	Austin	WQ5	-97.957	30.057	29	2	0.686	0.695	0.013	-
Water Quality Protection Lands	All	Austin	WQ	-	-	150	11	0.643	0.709	0.092	111

N, number of individuals collected at the location; N_p, number of private alleles; H_o, observed heterozygosity; H_e, expected heterozygosity; F_{IS}, inbreeding coefficient; N_e, effective population size.

Table S2. Characteristics of ten microsatellite loci amplified in 579 *Xylocopa virginica* females. Size range indicates allele DNA fragment length.

Locus	Multiplex Group	Size Range	N_A	H_o	H_e	F_{ST}	G'_{ST}
XV03	1	204-258	14	0.693	0.871	0.012	0.089
XV14	1	287-322	9	0.455	0.560	0.161	0.402
XV24	1	176-217	13	0.436	0.554	0.017	0.035
XV07	1	296-349	15	0.608	0.850	0.047	0.270
XV09	1	170-210	11	0.634	0.727	0.036	0.114
XV28	1	243-295	19	0.821	0.892	0.004	0.041
XF27	2	136-151	3	0.228	0.492	0.211	0.328
XF13	2	217-264	16	0.715	0.773	0.012	0.060
XG75	2	148-166	10	0.579	0.648	-0.018	-0.057
XV01	2	211-404	38	0.795	0.923	0.023	0.239
Global value			14.8	0.596	0.729	0.042	0.146

N_A , number of alleles; H_o, observed heterozygosity; H_e, expected heterozygosity; F_{ST}, fixation index (Weir & Cockerham 1984) ; G'_{ST}; genetic differentiation measure (Hedrick 2005). All F_{ST} and G'_{ST} values are significantly different from zero ($p<0.001$) aside from XG75 where negative values indicate no differentiation from zero.

Table S3. Datasets used for different analysis showing N genotypes included from each site. Analyses conducted on each dataset indicated by annotation (*, †, ‡, §)

			Dataset			
Site Name	City Region	Site Code	Original genotypes*	Full sibs removed (main dataset)†	20% missing data removed‡	Random individuals removed from transect sites§
Barton Creek Habitat Preserve	Austin	BC	55	52	34	29
Clymer Meadow Preserve	Dallas	CL	41	37	17	27
Clear Creek Natural Heritage	Dallas	CNH	7	7	7	7
Connemara Prairie	Dallas	CON	5	5	0	5
Harry S Moss Park	Dallas	HRM	9	9	8	9
Lake Whitney State Park	Waco	LKW	30	26	20	26
Mathew's Prairie	Dallas	MA	103	100	96	29
M & J Farms	Austin	MJF	10	9	8	9
Onion Creek Wildlife Sanctuary	Austin	ONC	5	5	5	5
Pedernales Falls State Park	Austin	PED	17	17	11	17
R & C Dairy	Dallas	RCR	9	9	7	9
Ray Roberts Lake State Park	Dallas	RRL	26	26	20	26
Smiley Woodfen Prairie	Dallas	SM	130	127	115	30
Water Quality Protection Lands	Austin	WQ	151	150	119	30
		Total	598	579	467	258

* Sibship

† Global F_{ST}, Global G'_{ST}, H_o, H_e, N_e, F_{IS}, IBD, IBR, Fine-Scale Spatial Auto-correlation

‡ Pairwise F_{ST} and G'_{ST}

§ Bayesian Genetic Clustering, Principal Components Analysis, Phylogenetic Tree

Table S4. Pairwise measures of genetic differentiation among the sampled sites. Pairwise G'_{ST} values are below diagonal, F_{ST} values are above diagonal. Significant pairwise differences are indicated in bold text ($\alpha < 0.05$). Significance is based on 9999 permutations in GenAlEx.

Population Code	BC	CL	CNH	HRM	LKW	MA	MJF	ONC	PED	RCR	RRL	SM	WQ
BC		**0.026**	**0.047**	**0.044**	**0.039**	**0.057**	0.025	0.039	0.014	**0.049**	**0.021**	**0.038**	**0.023**
CL	**0.070**		0.039	**0.039**	**0.036**	**0.040**	0.030	0.040	**0.032**	0.026	**0.026**	**0.018**	**0.024**
CNH	**0.113**	**0.057**		**0.061**	**0.060**	**0.051**	0.048	0.060	**0.057**	0.057	0.035	**0.038**	**0.033**
HRM	**0.102**	**0.065**	**0.115**		0.036	**0.081**	0.042	0.061	**0.050**	0.033	**0.045**	**0.061**	**0.052**
LKW	**0.136**	**0.114**	**0.179**	**0.050**		**0.086**	**0.037**	0.050	**0.050**	**0.042**	**0.050**	**0.061**	**0.049**
MA	**0.258**	**0.170**	**0.162**	**0.325**	**0.420**		**0.053**	**0.061**	**0.064**	0.058	**0.043**	**0.020**	**0.018**
MJF	**0.011**	**0.017**	**0.054**	**0.026**	**0.066**	**0.196**		0.043	0.028	0.044	0.032	**0.039**	0.021
ONC	**0.025**	**0.009**	**0.055**	**0.073**	**0.075**	**0.185**	**-0.037**		0.050	0.054	0.048	0.041	0.035
PED	**-0.018**	**0.055**	**0.122**	**0.086**	**0.152**	**0.259**	**-0.026**	**0.034**		**0.048**	0.027	**0.045**	**0.027**
RCR	**0.127**	**-0.027**	**0.089**	**-0.051**	**0.080**	**0.214**	**0.025**	**0.009**	**0.071**		**0.040**	**0.043**	**0.041**
RRL	**0.045**	**0.056**	**0.038**	**0.094**	**0.183**	**0.178**	**0.037**	**0.063**	**0.031**	**0.064**		**0.027**	**0.022**
SM	**0.171**	**0.047**	**0.096**	**0.232**	**0.305**	**0.099**	**0.122**	**0.066**	**0.170**	**0.132**	**0.104**		**0.018**
WQ	**0.092**	**0.090**	**0.066**	**0.181**	**0.229**	**0.090**	**0.010**	**0.034**	**0.069**	**0.124**	**0.072**	**0.097**	

Table S5. MRDM results investigating geographic distance, and resistance distances for all land-use resistance surfaces (Hypotheses A, B, and Set C, 20 total resistance models) and their relationship to average Bruvo's genetic distance per site (N=528 pairs). Significant models are highlighted in bold.

		Resistance Value per Land Cover Type					MRDM Results		
Hypothesis and Model Number	Landcover classes set to Lower Resistance (0.1-0.3)	Developed	Cultivated	Forest & Shrub	Grassland	Other Classes*	F	R^2	p-val
Geographic Distance Model 1 (all sites included)	NA	NA	NA	NA	NA	NA	**15.561**	**0.028**	**0.009**
Geographic Distance Model 2 (sites >1 km apart)	NA	NA	NA	NA	NA	NA	**4.011**	**0.023**	**0.042**
A Model 1	Grassland & Forest	0.9	0.9	0.1	0.1	0.9	4.265	0.008	0.256
A Model 2		0.5	0.5	0.3	0.3	0.5	**24.517**	**0.045**	**0.003**
A Model 3		0.7	0.7	0.1	0.1	0.7	5.923	0.011	0.163
A Model 4		0.9	0.9	0.3	0.3	0.9	**15.116**	**0.028**	**0.015**
B Model 1	Developed, Cultivated & Forest	0.1	0.1	0.1	0.9	0.9	**19.452**	**0.035**	**0.008**
B Model 2		0.3	0.3	0.3	0.5	0.5	**31.569**	**0.057**	**<0.001**
B Model 3		0.1	0.1	0.1	0.7	0.7	**21.789**	**0.040**	**0.005**
B Model 4		0.3	0.3	0.3	0.9	0.9	**28.555**	**0.051**	**0.002**
Set C Model 1	Developed only	0.1	0.9	0.9	0.9	0.9	**18.114**	**0.033**	**0.017**
Set C Model 2		0.3	0.5	0.5	0.5	0.5	**31.737**	**0.057**	**0.001**
Set C Model 3		0.1	0.7	0.7	0.7	0.7	**20.852**	**0.038**	**0.006**
Set C Model 4		0.3	0.9	0.9	0.9	0.9	**28.772**	**0.052**	**0.001**
Set C Model 5	Cultivated only	0.9	0.1	0.9	0.9	0.9	**38.810**	**0.068**	**0.001**
Set C Model 6		0.5	0.3	0.5	0.5	0.5	**39.617**	**0.070**	**<0.001**
Set C Model 7		0.7	0.1	0.7	0.7	0.7	**40.303**	**0.071**	**0.001**
Set C Model 8		0.9	0.3	0.9	0.9	0.9	**43.006**	**0.076**	**<0.001**
Set C Model 9	Forest only	0.9	0.9	0.1	0.9	0.9	8.111	0.015	0.109
Set C Model 10		0.5	0.5	0.3	0.5	0.5	**25.460**	**0.046**	**0.003**
Set C Model 11		0.7	0.7	0.1	0.7	0.7	9.859	0.018	0.070
Set C Model 12		0.9	0.9	0.3	0.9	0.9	**17.891**	**0.033**	**0.008**

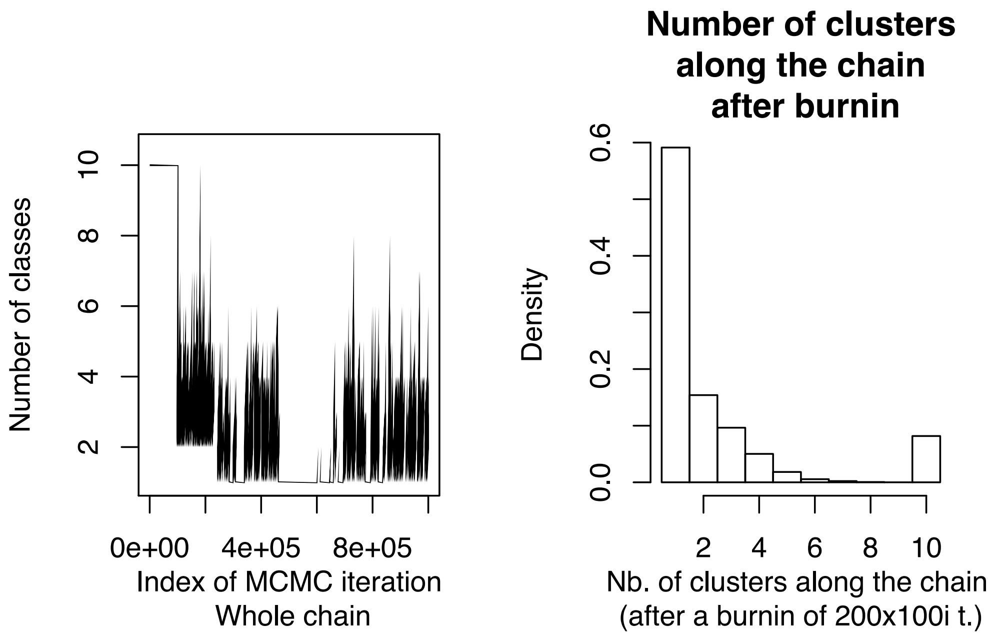

Figure S1. Plots showing the likely number of genetic clusters inferred by the Bayesian clustering program GENELAND (Guillot *et al.* 2005). Both plots show a clear mode at K=1 across 1,000,000 Markov Chain Monte Carlo runs.

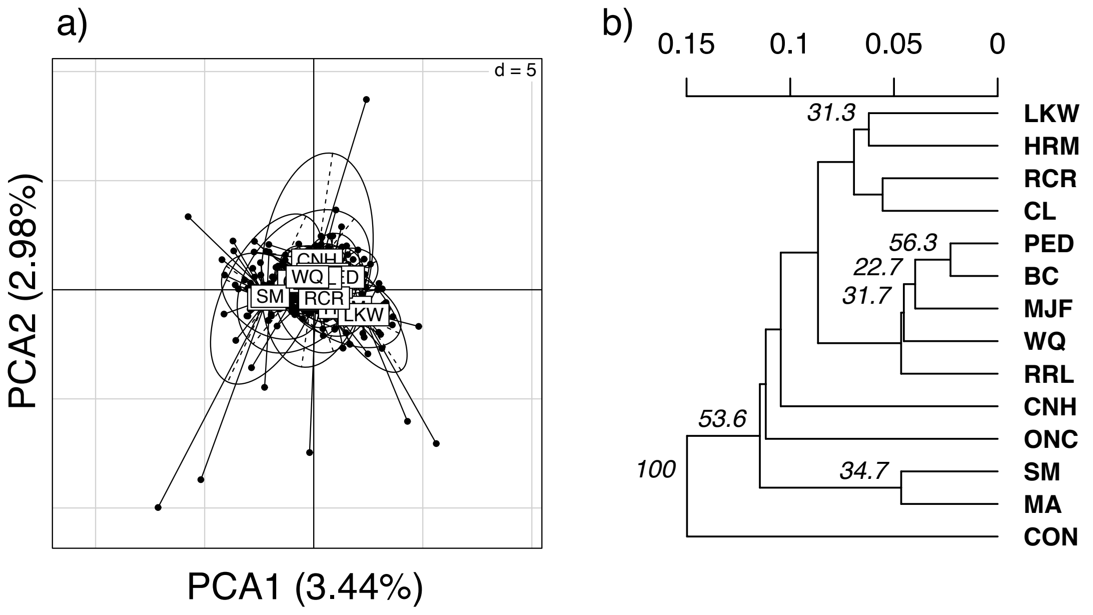

Figure S2. Additional genetic clustering analyses using a maximum of 30 individuals per site (Site codes defined in Tables S1 and S3). a) Principal components analysis of individual genetic differentiation between sites showing the first two PC axes. b) Rooted dendrogram with 1000 bootstraps using Nei's genetic distance between sites. Node labels indicate percentage of trees supporting node differentiation, nodes with less than 20% support are not labelled. Branch lengths (top scale) estimated with Nei's distance.
